# Diverse Biological Functions of Sphingolipids in the CNS: Ceramide and Sphingosine Regulate Myelination in Developing Brain but Stimulate Demyelination during Pathogenesis of Multiple Sclerosis

**DOI:** 10.13188/2332-3469.1000035

**Published:** 2017-12-23

**Authors:** Somsankar Dasgupta, Swapan K. Ray

**Affiliations:** 1 Department of Neuroscience and Regenerative Medicine, Augusta University, USA; 2 Department of Pathology, Microbiology, and Immunology, University of South Carolina School of Medicine, USA

**Keywords:** Ceramide, Central nervous system, Cytokines, Demyelination, Developing brain, myelination, Sphingolipids, Sphingosine

## Abstract

Sphingolipids are enriched in the Central Nervous System (CNS) and display multiple biological functions. They participate in tissue development, cell recognition and adhesion, and act as receptors for toxins. During myelination, a variety of interactive molecules such as myelin basic protein, myelin associated glycoprotein, phospholipids, cholesterol, sphingolipids, etc., participate in a complex fashion. Precise roles of some sphingolipids in myelination still remain unexplored. Our investigation delineated participation of several sphingolipids in myelination during rat brain development as well as in human brain demyelination during pathogenesis of Multiple Sclerosis (MS). These sphingolipids included Ceramide (Cer)/dihydroceramide (dhCer), Sphingosine (Sph)/dihydrosphingosine (dhSph), and glucosyl/galactosylceramide (glc/galCer) as we detected these by column chromatography, high performance thin-layer chromatography, gas chromatography-mass spectrometry, and high-performance liquid chromatography. Cer/dhCer level rises during rat brain development starting at Embryonic stage (E) until postnatal day (P21), then gradually falls until the maturity (P30 and onwards), and remains steady maintaining a constant ratio (4–4.5:1) throughout the brain development. GlcCer is the initial Monoglycosylceramide (MGC) that appears at early Postnatal stage (P8) and then GalCer appears at P10 with an increasing trend until P21 and its concentration remains unaltered. Sph and dhSph profiles show a similar trend with an initial peak at P10 and then a comparatively smaller peak at P21 maintaining a ratio of (2–2.5:1) of Sph:dhSph. The profiles of all these sphingolipids, specifically at P21, clearly indicate their importance during rat brain development but somewhat unspecified roles in myelination. While Cer has been reported to involve in neurodegenerative diseases such as Alzheimer’s disease and Parkinson’s disease, Sph being a potent inhibitor of protein kinase C has recently been implicated in CNS demyelination due to MS. Inflammatory cytokines stimulate Sph elevation in MS brains and lead to demyelination due to oligodendrocyte death as we examined by using human oligodendroglioma culture. In conclusions, sphingolipids are essential for brain development but they have deleterious effects in demyelinating diseases such as MS.

## Introduction

Sphingolipids are classified as a group of lipids that contain either a Sphingosine (Sph) or dihydrosphingosine (dhSph) base with a fatty acyl group often attached to the second carbon (C2) atom via -NHCOR linkage (designated as ceramide or Cer, where R represents the fatty acyl chain) [1]. Sphingolipids, which include over 4000 bioactive lipid molecules, are significant players in multiple biological processes such as signal transduction, stress responses, immune reaction, membrane structure, and brain development. Sphingolipids containing one or more carbohydrates are synthesized by addition of carbohydrates to the first Carbon (C1) of Cer, where the first carbohydrate is usually Glucose (Glc) or Galactose (Gal) and they are known as Glycosphingolipids (Gsls) [2]. Gsls have been extensively studied for their variegated functions in the body such as cell development and growth, differentiation, antigen receptor, cell-cell recognition, protein binding, cancer cell death (apoptosis), and inborn errors in lipid metabolism [3–10]. Cer is now at the center of many studies for its unique participation in many cellular events including its devastating role in human nervous diseases such as Alzheimer’s disease (AD) and Multiple Sclerosis (MS) while Sph has long been indicated as an inhibitor of Protein Kinase C (PKC) [11–14]. Exploration of complex roles of sphingolipids in AD and MS is an active field of investigation. One such recent investigation demonstrated the role of Sph in neurodegeneration in the Central Nervous System (CNS) [15]. However, the roles of Cer and Sph/dhSph in CNS development leading to myelination and maturity have not yet been examined.

GlcCer is the key component of all long-chain Gsls starting from lactosylceramide while GalCer is the major component of myelin. Its importance in myelination has been unequivocally established by studying the Cer:galactosyltransferase (GalT1) knockout mice [16]. The GalT1 knockout mice survive until the day of maturity and then develop seizures leading to death [16]. Although there is increase in GlcCer in myelin, the lack of galactosylceramide plays a detrimental but unspecified role in myelin structural and functional integrity, which cannot be compensated by GlcCer. However, it is noteworthy that the precise mechanism of GalCer in myelination still remains obscure. Moreover, the regulation of Sph/dhSph and Cer during the brain development and myelination has never been reported.

Although the Sph toxicity as an inhibitor of PKC has been established [14], its role in neurological disorder has not been reported until a recent report from our laboratories indicating Sph toxicity in degeneration of oligodendrocytes and neurons in MS demyelination [15]. In this manuscript, we report the regulation of three sphingolipids such as Cer, Sph, and dhSph in CNS development and myelination using a rat model. In support of our theme for this manuscript, we also describe the involvement of two other Monoglycosylceramides (MGCs) such as GlcCer and GalCer during CNS development and myelination to further demonstrate the devastating role of Sph in CNS demyelination in course of MS [17].

## Materials and Methods

### Animals and chemicals

Pregnant albino rats were purchased from Charles River Laboratories (Wilmington, MA) and the mother and the pups were sacrificed under anesthesia to collect the Embryonic (E) and Postnatal (P) brains following the protocol in accordance with the Guide for the Care and Use of Laboratory Animals from the US Department of Health and Human Services (National Institutes of Health, Bethesda, MD), as approved by the Institutional Animal Care and Use Committee (IACUC) at the Medical University of South Carolina (Charleston, SC). Brain tissues from pups at the different growth period starting from Postnatal day 1 (P1) to Postnatal day 30 (P30) and adult animals were collected and preserved at −80°C until use. Solvents were purchased from Fisher Scientific (Hampton, NH), resins were purchased from Pharmacia LKB Technology (Uppsala, Sweden), high performance thin-layer plates were obtained from EM Science (Gibbstown, NJ), silicic acid and standard sphingolipids were obtained through Sigma-Aldrich (St. Louis, MO). Human Oligodendroglia (HOG) cell line was a generous gift from Prof. Robert K. Yu, Department of Neuroscience and Regenerative Medicine, IMMAG, Augusta University (Augusta, GA). All other reagents and chemicals used in our experiments were of analytical grades.

### Animal studies

Purification of Ceramide (Cer), Monoglycosylceramides (MGCs), Sphingosine (Sph), and dihydrosphingosine (dhSph) from rat brain: Total brain lipids were extracted using chloroform-methanol mixture and Cer, Sph/dhSph, and MGCs were purified as described using a silicic acid (0.5 × 22 cm) column [17]. Briefly, The lipid extracts were applied on the column using chloroform:acetone (1:1, v/v) and washed with the 10–15 column volume of the same solvent. Cer was eluted as a single fraction using choloroform:acetone (90:10, v/v), MGCs were eluted using chloroform:methanol (23:2, v/v) while all lipids including Sph bases were eluted from the column using tetrahydrofuran:water (7:1, v/v). Sph/dhSph elution was further purified using a Dowex 50X8-200 (Na+ form) column, washed and the bases were eluted with methanol containing 0.4 M CaCl_2_. The eluent was dried and salt was removed by using a Sep-Pak C18 cartridge [18].

#### Thin layer chromatography of ceramide and monoglycosylceramides

Cer purified by silicic acid chromatography was dissolved in a define volume - (1 ml/g of tissue) of chloroform:methanol (9:1, v/v) and 15 μl was applied on a High Performance Thin Layer Chromatography (HPTLC) plate, individual bands were resolved using chloroform:methanol:acetic acid (95:4.5:0.5, v/v/v) and visualized using benzidine spray [17,19]. Each band was scanned using Image J program, compared to standard Cer bands, and quantified.

MGCs fraction was dried and dissolved in a defined volume (1 ml/mg for embryonic to P10 and 2 ml/g from P15 to adult) of chloroform: methanol (9:1, v/v) and was applied quantitatively (5 μl) on a HPTLC plate pre-coated with 1% boric acid. The bands were resolved using chloroform: methanol: 0.1 M boric acid (65:25:3, v/v/v) and visualized by diphenylamine aniline spray [20]. Bands from each lane were scanned and quantitated using Image J program and compared to reference standard (GalCer and GlcCer).

#### Structural elucidation of Cer, MGCs, and Sph by Gas Chromatography and Mass Spectrometry (GC-MS)

A portion of the Cer solution (10 μl) was removed, dried and methanolyzed with methanol:water:HCl (29:4:3, v/v/v) at 80°C for 18 h. The solution was washed with an equal volume of hexane (3 times) to remove partially methylated fatty acids. The recovered fatty acids were further methylated with 1 N methanolic HCl and recovered using hexane. Methylated fatty acids were analyzed as methyl derivative while the lower phase of the partial methylation was neutralized, recovered in ether, and analyzed as the Trimethylsilyl (TMS) derivative in a Hewlett-Packard GC-MS (GC 5980, MS 5972) [21].

A portion of the purified MGCs solution was preserved, permethylated, hydrolyzed, and acetylated and the carbohydrate content was analyzed by GC-MS as permethylated alditol acetates on a DB-1 column [20]. Fatty acids and base compositions (TMS derivative) of MGCs were analyzed by GC-MS as described previously. The base composition of the MGCs was further analyzed by High Performance Liquid Chromatography (HPLC) after fluorescent tagging as described below.

#### High performance liquid chromatography (HPLC) for Sph bases

Cer, bases from MGCs and the purified Sph extracts were assayed for the Sph bases by HPLC (Waters; Milford, MA) after fluorescent tagging [22,23]. The bases of the Cer extracts (collected after methanolic hydrolysis) and MGCs were hydrolyzed, fatty acids were analyzed by GC-MS, and bases were analyzed by HPLC after tagging as well as by GC-MS as trimethylsilyl derivatives [21]. Cer was also assayed by HPLC as the 3-keto derivative [24].

### Human tissue studies

#### Ceramide, MGCs, and sphingosine content of the Multiple Sclerosis (MS) brain

Six individual sets of normal brain tissues (persons died by accident) and tissues from MS patients were collected from National Neurological Research Specimen Bank (Los Angeles, CA). Brains were carefully dissected into Normal Appearing White Matter (NAWM) and plaque. Approximately, 30–50 mg of tissue were dissected and preserved at −80°C before using. Cer, MCGs, and Sph were isolated from NAWM and plaque area and each component was quantitated employing HPTLC, GC-MS, and HPLC as described above [25,26].

### Cell culture studies

#### Determination of cellular toxicity of Sph in the cultured oligodendrocytes (HOG cells)

HOG cells were cultured in two 96-well plates (approx.10,000–12,000 cells/well) and incubated for 48 h. Cells were treated with Sph at various concentrations in DMEM containing 0.2% Fetal Bovine Serum (FBS) and 0.04% Dimethyl Sulfoxide (DMSO) and incubated for 24 h. Stock solutions of DMEM, 0.2% FBS, and 0.04% DMSO (Media A) were prepared and used for dilution. A Sph stock solution (40 μM) was prepared by evaporating 20 μl of 10 mM Sph (in ethanol), adding 2 μl of DMSO (0.04%), and mixing with 5 ml of the media containing 0.2% FBS (stock). The stock solution was diluted to a range of 0.25 μM to 30 μM concentrations. Two sets of control were used, one containing the regular media with 10% FBS and the second one cultured in media A. Cells were incubated for another 24 h and the cell viability was measured using cell counting kit 8 (Dojindo Molecular Technologies, MD) [15].

### Statistical analysis

Animal experiments for studying myelination were repeated 2 times. Both experiments showed almost identical results. Considering the cost and the lengthy procedure but accuracy of results, we did not repeat a third set. Six human tissues (control and MS) were used for sphingolipid analysis. Analysis of Variance (ANOVA) with Tukey’s post-hoc analysis was used to compare the different groups. Results were shown as Mean ± Standard Deviation (SD). All p values reported were two sided and a p<0.05 was considered statistically significant. All statistical analysis was performed using MS Excel v2010 or R (V3) on a Windows XP platform.

## Results

### Developmental profile of Ceramide (Cer) in rat brain

**Figure 1:**
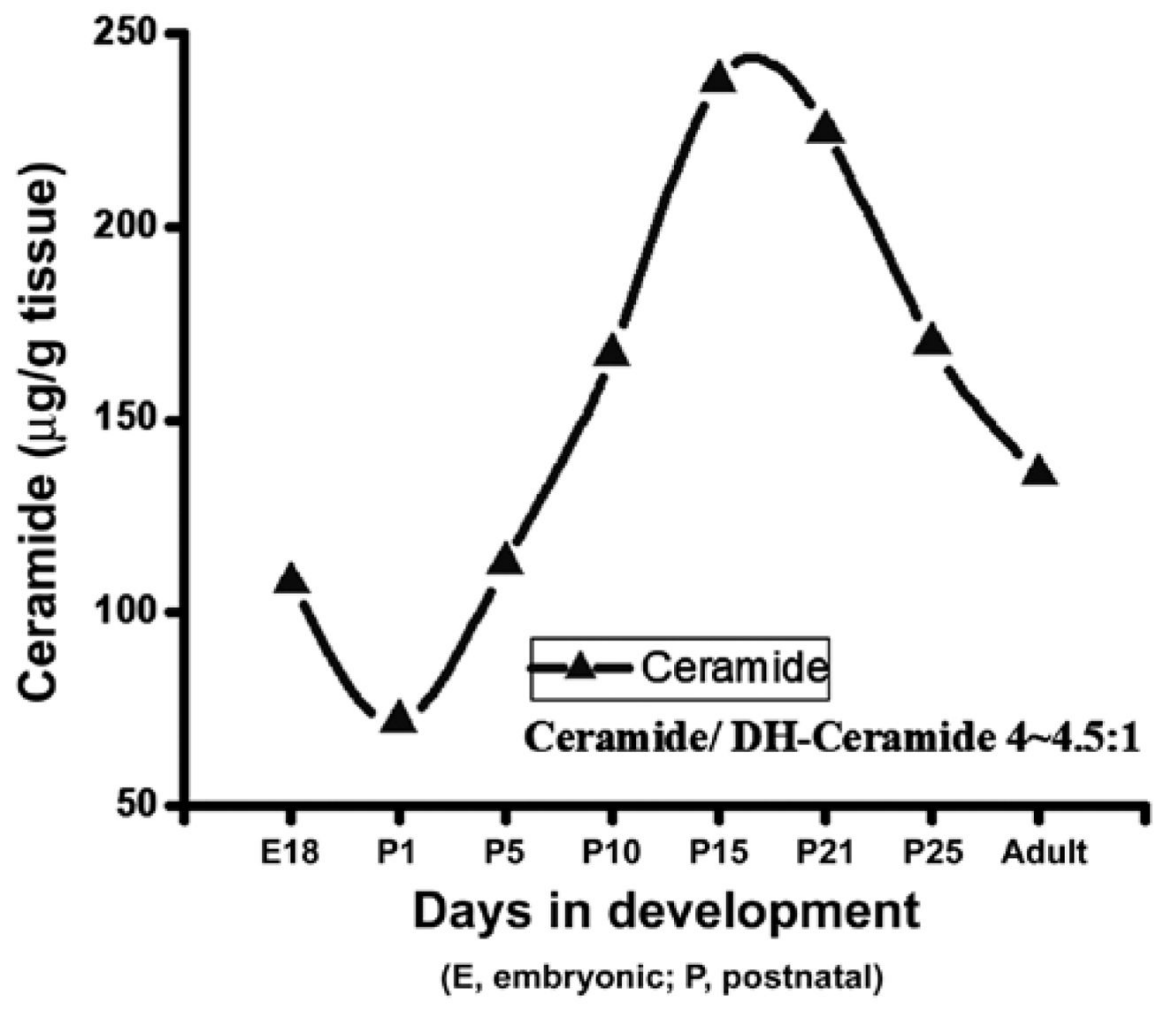
Evaluation of ceramide synthesis during rat brain development. Ceramide fractions were purified by column chromatography and applied quantitatively on HPTLC. The individual bands were resolved and quantified using the Image J program. Ceramide structure was delineated by GC-MS and followed by HPLC and the sphingosine/dh sphingosine content was determined. E: Embryonic brain; P: Postnatal brain. The curve represents results obtained using two sets of animals.

Cer level was examined in developing rat brain tissues after purification and HPTLC resolution. Its concentration was determined using scanning and the composition (fatty acids and base) was determined by GC-MS and also quantified by HPLC. Our data clearly indicated that brain Cer level was gradually elevated to approximately 235–245 μg/g starting at embryonic stage until the P21 (peak for myelination) and then reduced to a certain level (170–175 μg/g) until P30 and remained nearly same until the adult stage (Figure 1). The increasing concentration of Cer after an initial dip at E18 (starting at 105–110 μg/g at E18, down to 70–75 μg/g at P1, and then elevated to 235–245 μg/g at P21) during the brain development along with a peak at the critical stage of the myelination of axons clearly signifies its importance in myelination in developing rat brain. It is interesting to note that the ratio of Cer/dhCer (measured by Sph/dsph content) stays at 4.0–4.5/1 (in both sets of experiment) throughout the brain development in rats.

### Concentration of MGCs increased in developing brain in rats-conversion from Glucosylceramides to Galactosylceramides during myelination

**Figure 2:**
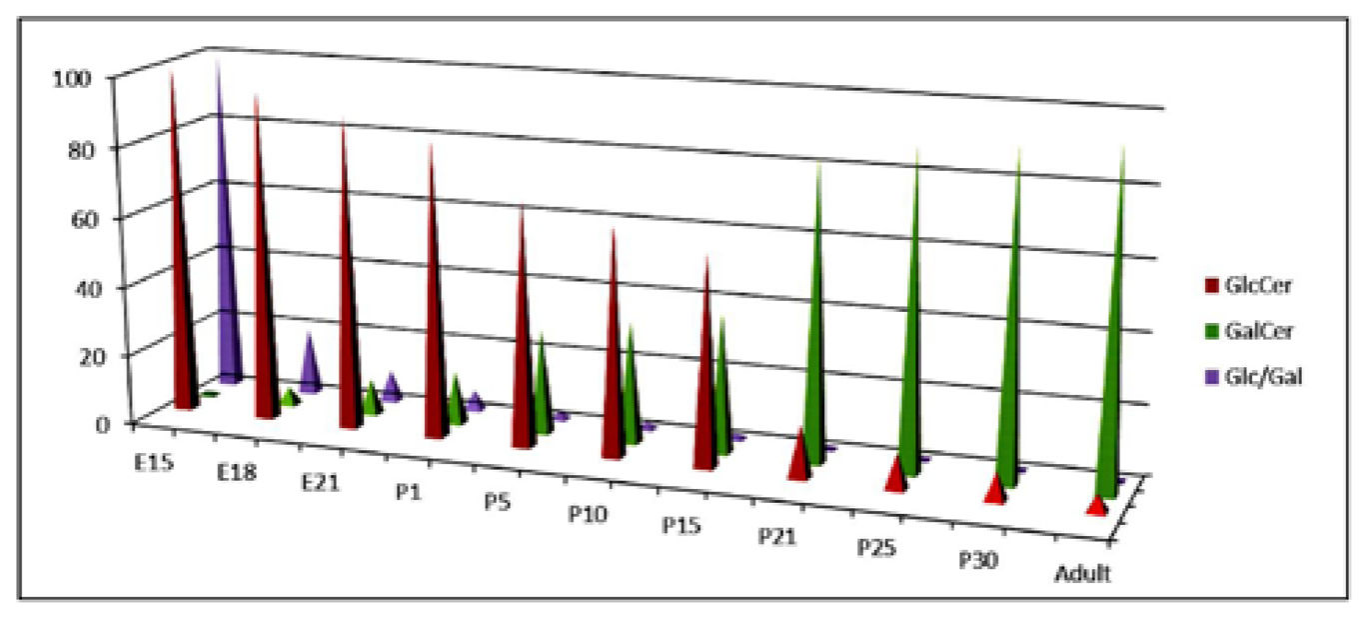
Monoglycosylceramide content in developing rat brain. Monoglycosylceramide fraction was purified by column chromatography and the individual bands were resolved using HPTLC. Each band was quantified using the Image J program and the structure was elucidated using GC-MS. GlcCer: Glucosylceramide; GalCer: Galactosylceramide; E: Embryonic brain; P: Postnatal brain.

The composition of purified MGCs was identified and quantified by HPTLC. The structural characterization (both carbohydrate and base compositions) and quantitation of MGCs (GlcCer, GalCer, and Glc/Gal) were performed using GC-MS. During the embryonic development and at early postnatal stage, GlcCer was the only MGC identified in the rat brain until P8 (Figure 2). GlcCer concentration was at the highest level at E15, gradually decreased until P15, and then dramatically dropped culminating to a negligible level at adult stage. On the other hand, GalCer concentration was gradually increased from E18 up to an optimal level of expression at P21 and then remained almost unchanged until the adult stage. The carbohydrate composition was determined by GC-MS and the developmental profile of carbohydrate along with the Glc/Gal ratio was also determined (Figure 2). The Glc/Gal ratio was found to be the highest at E15 and the lowest at adult rat brain. Moreover, we determined the Cer:UDP glucosyltransferse and Cer:UDP galactosyltransferase activities. It is noteworthy that the Glc/Gal ratio is well correlated with the Cer:UDP glucosyltransferse and Cer:UDP galactosyltransferase activities.

### Elevation of Sph and dhSph levels during brain development and their reduction there after

**Figure 3:**
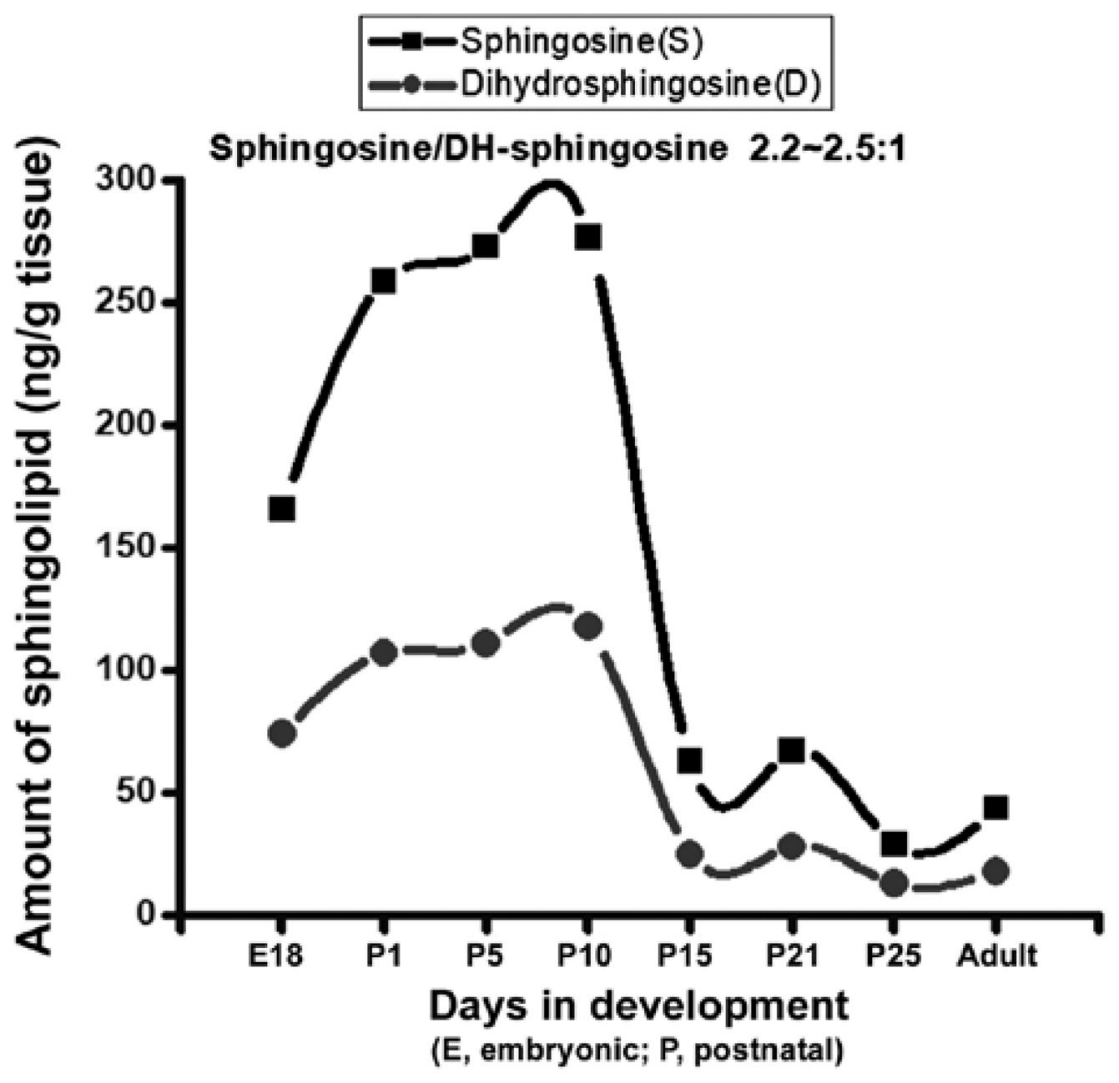
Sphingosine and dihydrosphingosine contents in rat brain during development. Sphingosine fractions were purified by column chromatography and quantified using HPLC and GC-MS. E: Embryonic brain; P: Postnatal brain. The curve represents results obtained using two sets of animals..

We purified Sph and dhSph fractions from the brain tissues and analysed by HPLC and GC-MS after formation of trimethylsilyl derivatives. Both Sph and dhSph levels were found to increase during brain development starting from embryonic stage (160–165 ng/g and 70–75 ng/g, respectively at E18) and reached their peaks at P10 (280–290 ng/g and 120–125 ng/g respectively) and then gradually decreased (50–55 ng/g and 15–20 ng/g, respectively) (Figure 3). It is worth mentioning that the Sph and dhSph levels reached relatively low second peaks (60–65 ng/g for Sph and 25–30 ng/g for dhSph) at P21 that coincided with the peak of myelination in the rat brain. Another interesting observation was that like Cer, a constant ratio of Sph/dhSph was always maintained at 2.2–2.5/1 in the normal brain during its development.

### Increase in Sph level while decreases in Cer and MGCs levels in MS brain

**Figure 4:**
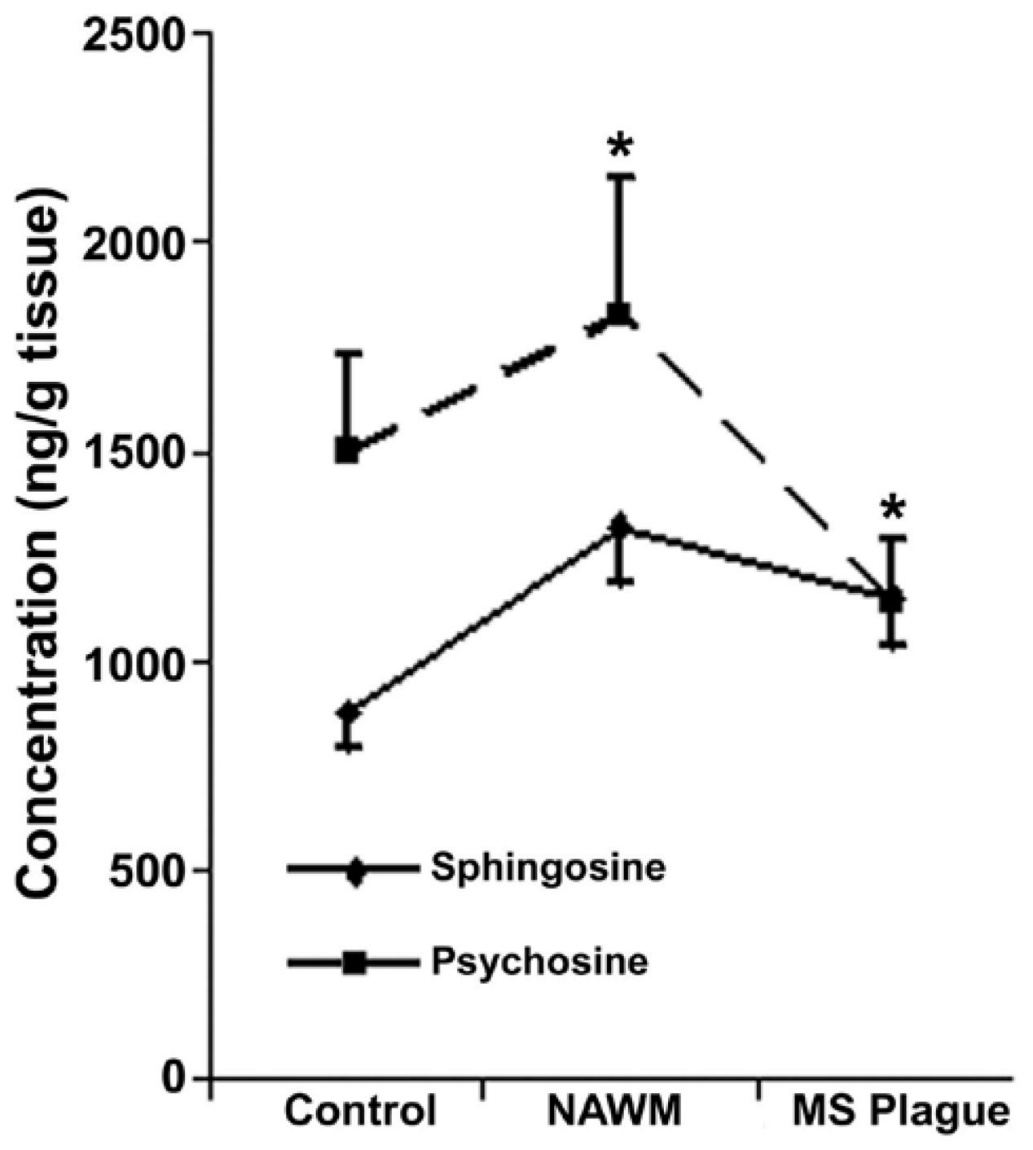
Sphingosine and psychosine contents in normal human and MS brains. Human MS brain was dissected as normal appearing white matter (NAWM) and plaque. A similar region was dissected from normal brain. Sphingosine and psychosine fractions from normal and MS brain tissues were purified using column chromatography and quantified using HPLC and GC-MS. The data were presented as mean±SD of six individual sets of brain. An (*) asterisk indicates a p value of <0.05.

Content of sphingolipids such as Cer, MGCs, and sphingoids were analysed in six control and six MS brain tissues (Figure 4). Sphingoid content, specifically Sph, was increased by 1.5- to 1.75-fold in MS plaques while psychosine (galactosyl-Sph) was increased in NAWM but reduced to 0.5-fold of the control in the plaque(an asterisk indicates a significant p value). Psychosine, another toxic but a minor sphingolipid of myelin, showed an increase in NAWM but decrease in MS plaque. A reduction in MGCs (GalCer and fast migrating cerebrosides or FMCs) content of NAWM (0.65-fold) and plaque (0.5-fold; data not shown) was consistent with demyelination because the two components of myelin [27], MGCs and psychosine, were previously found to decrease in myelinolytic disorders [27]. Total Cer level showed a decreasing trend in NAWM (0.75-fold) and MS plaque (0.65-fold; data not shown). It is anticipated that Cer may be converted to Sph by ceramidase and partially lost during active demyelination. However, we postulate that the Cer level is transiently increased during early MS progression and this event has been demonstrated to occur using EAE animals [15]. Its level was reduced after the onset of demyelination.

### Sph-mediated cell death in oligodendrocytes

**Figure 5:**
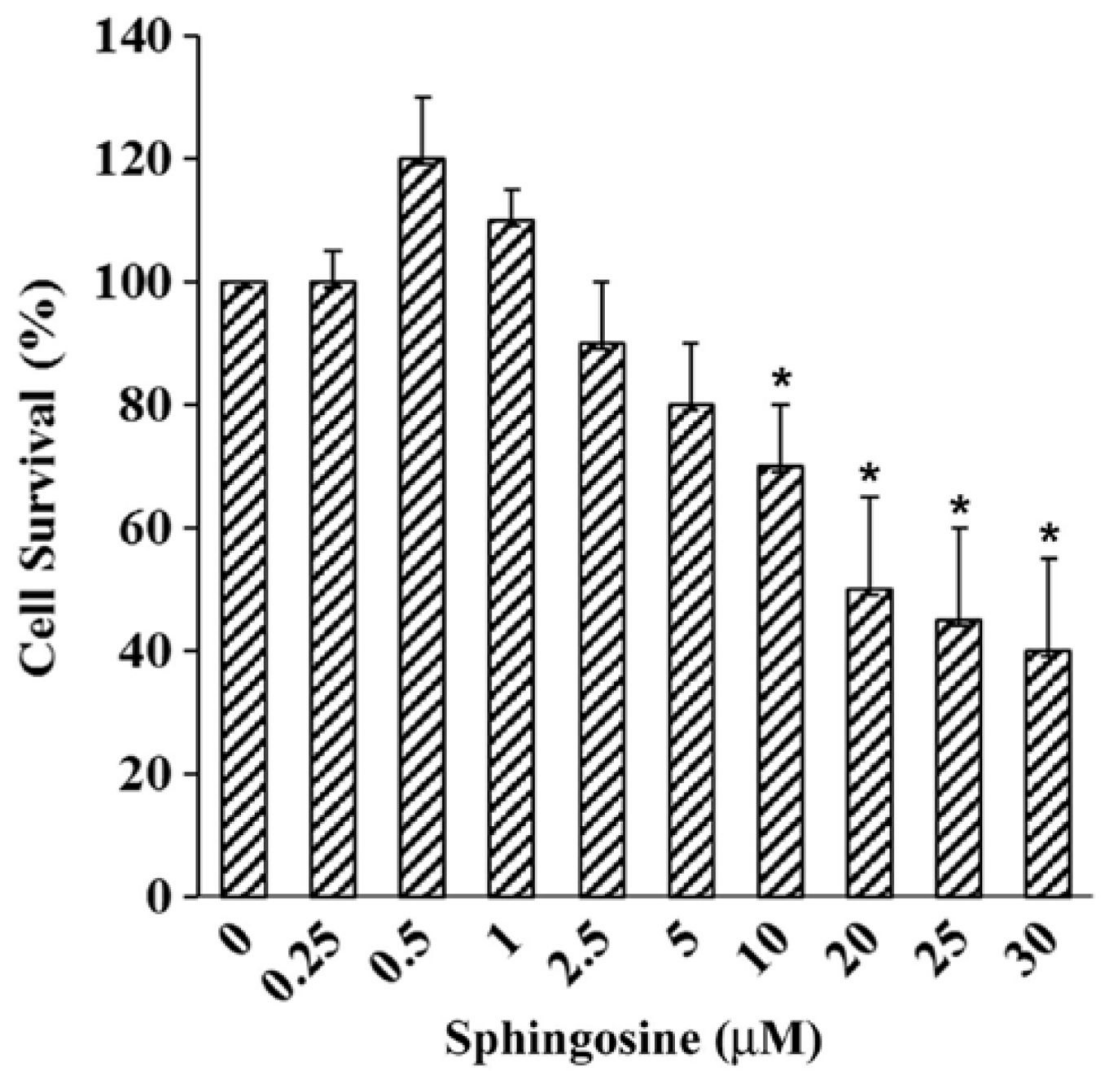
Oligodendrocyte HOG cell death due to treatment with sphingosine. HOG cells were cultured and incubated with various concentration of sphingosine. Cells were incubated for 24 h and the viability was measured using cell-counting kit. An initial stimulation of growth of the cultured HOG cells was observed at a low concentration of sphingosine while a higher concentration of sphingosine stimulated cell death. The bar graph represents a set of three individual experiments. An asterisk (*) indicates a p value of <0.05.

Sph is a minor component of normal brain and we have observed an accumulation of Sph in MS brain. Sph mediates cell death by inhibiting PKC [14]. To test whether Sph toxicity could mediate apoptosis in oligodendrocytes, Human Oligodendroglioma (HOG) cells were exposed to different concentrations of Sph (Figure 5). Cells were cultured in media containing DMEM and Fetal Bovine Serum (FBS) for 48 h. The cells were then treated with Sph in presence of 0.2% FBS and further incubated overnight (15–18 h) and the cell viability was measured. An asterisk indicates a significant p value. A gradual decrease in HOG cell growth was observed with Sph at concentrations between 2.5–30 μM. A mild increase in cell growth at Sph concentration between 0.5–1.0 μM supported the notion that Sph at a lower concentration mediated oligodendrocyte cell growth via TRPM3 activation [28]. This is further supported by our observation (in animal model) that Sph concentration increases during brain development at an early stage of oligodendrocyte generation (280–290 ng/g at P10) and also during peak myelination at P21 (60–65 ng/g) (Figure 3).

## Discussion

Biological functions of sphingoids (sphingosine, dhsphingosine, and psychosine) and sphingolipids are pertinent to their relative concentrations in cells and tissues [27]. Hence, it is important to determine their precise concentrations in order to correlate their biological functions. For example, Cer and other sphingolipids metabolites participate in a wide variety of biochemical events such as protein phosphorylation, modulation of PKC and phospholipase A2 [1], and mediation of signal transduction leading to cell death while Sph and psychosine are potent inhibitors of PKC [1,11,14,29–32]. All these functions are relevant to the in situ lipid concentrations. Although the precise concentration of a sphingolipid metabolite is the key to normal tissue development and maintenance, the normal concentrations of Cer and Sph in normal tissue growth and function have never been examined.

We, for the first time, examined the Cer, Sph/dhSph, and MGCs (GlcCer and GalCer) concentrations in normal rat brain development. Employing a method developed in our laboratory [17], we purified, characterized, and quantified the concentrations of Cer, Sph/dhSph, and MGCs in order to evaluate their functions in brain development and myelination. Our study indicates that all three components are critically important for brain development and myelinogenesis. An increasing concentration of Cer during the growth with an optimum concentration at P21 clearly signifies its importance in myelination although the precise mechanism (i.e. interactions with other myelin components) has yet to be explored. It is likely that Cer being the core structure, it may stimulate the synthesis of other sphingolipids such as GalCer, GM1, etc. that are pertinent interactive biomolecules to myelination. Here, we have quantitated the Cer/dhCer ratio using GC-MS and HPLC during the rat brain development to report that Cer/dhCer ratio always maintains a constant value between of 4.0–4.5/1. A great variation of this ratio has been observed in MS brain where an aberrant sphingolipid metabolic activity has recently been confirmed [15].

MGCs have been shown to have a unique expression during development [20,27]. A detail fatty acid developmental profile of the two MGCs, GlcCer and GalCer, during brain development has also been reported [20]. Of MGCs, GlcCer is the only component in embryonic and early postnatal brain (P2–P5) and then during subsequent developmental stages (P8–P10) brain initiates GalCer synthesis with gradual increasing concentration, peaks at P21 and then stabilizes (Figure 2). We have detected GlcCer in the adult rat brain but its concentration is negligible when we have compared to GalCer concentration. The activities of two enzymes, Cer:UDP glucosyltransferase and Cer:UDP glucosyltransferase were in compliance with the GlcCer and GlaCer concentrations [20]. A large decrease in GalCer in MS brain tissue has also been reported as a direct manifestation of extensive demyelination [27].

**Figure 6:**
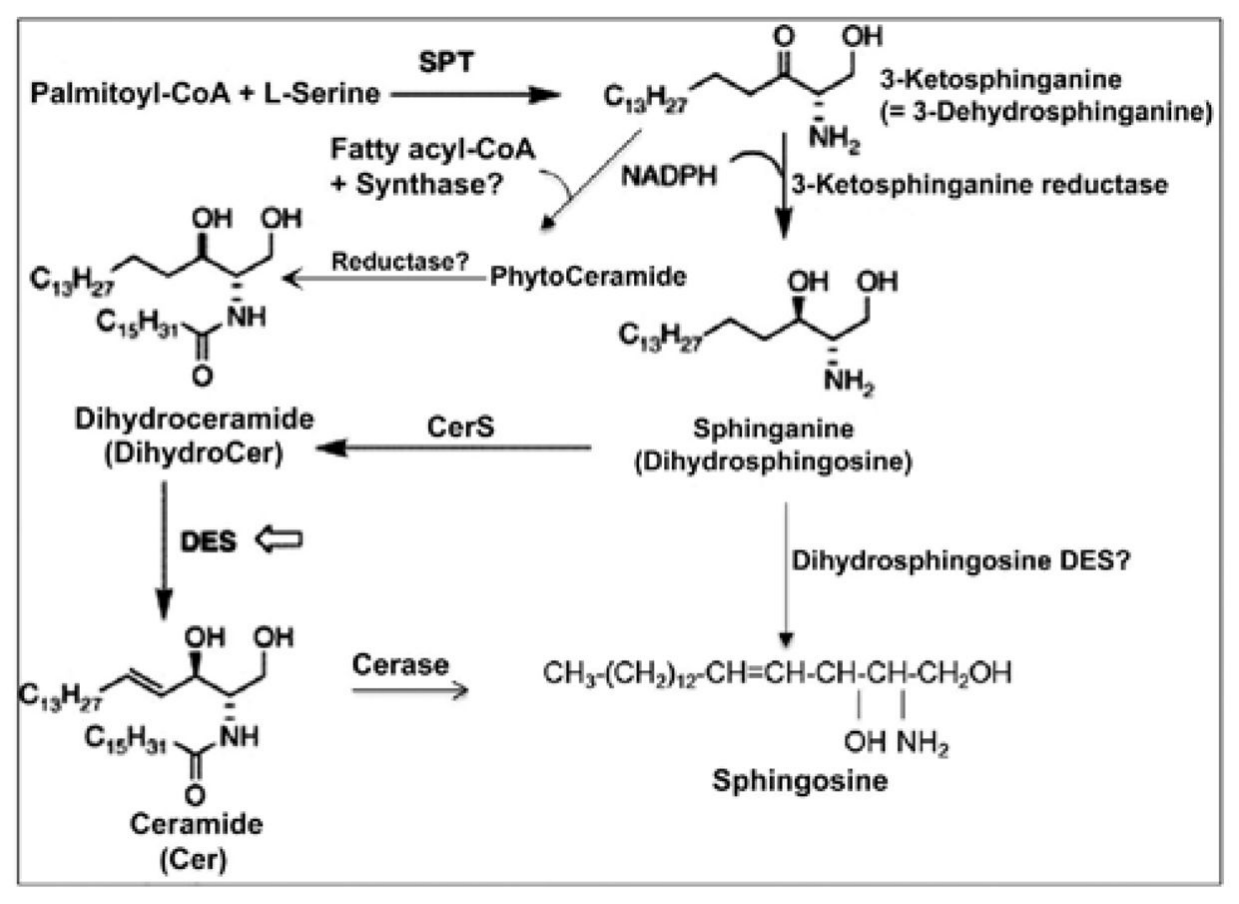
Ceramide and sphingosine biosynthesis in vertebrate. Cerase: Ceramidase; CerS: Ceramide synthase; DES: Desaturase; SPT: Serine palmityoltransferase.

Similarly, Sph/dhSph also plays a unique role in myelination as their concentrations are gradually elevated until P10 and then decreased. It shows another short peak during the myelination at P21 with a steady ratio of Sph/dhSph is 2.2–2.5/1. Again, this ratio shows a great variation in MS brain. Cer biosynthesis is mediated via dhCer (by desaturase), which in turn is converted to Sph (Figure 6). We anticipate that the accumulation of Sph may be triggered by excess Cer biosynthesis (due to break down of Cer by ceramidase) as shown in the biosynthetic diagram (Figure 6). Sph has an unknown but critical role in oligodendrocyte synthesis during myelination by activating TRMP3 [28]. Hence, this may be reflected by the appearance of the peak at P10 and then at P21 correlating with the activation of TRMP3 during myelination as TRPM3 participates as a Ca2+-permeable and Sph-activated channel in oligodendrocyte differentiation and CNS myelination (Figure 3) [28]. In addition, we have observed that a lower Sph concentration stimulates HOG cell proliferation while a higher concentration leads to cell death (Figure 5). However, the detail mechanism by which Cer and Sph aids myelinogenesis remains to be explored.

An early report by Dasgupta et al. described the chromatographic purification and characterization of phyto Cer in vertebrate brain and other tissues indicating that a reevaluation of Cer biosynthesis might be warranted since no such Cer species was previously reported to occur naturally in vertebrate tissues [33]. We postulate that phyto Cer may be biosynthesized by addition of a fatty acyl group to phyto Sph by a phyto Cer synthase and, to the best of our knowledge, this enzyme has not yet been reported (Figure 6). Besides, phyto Cer could possibly be a transient component as it might lead to the biosynthesis of dhCer by a novel reductase (Figure 6). Moreover, a huge accumulation of Sph at an initial developmental stage (P10) without any peak for Cer at this stage makes us wonder whether a Desaturase (DES) may exist which directly converts dhSph into Sph and again the existence of such an enzyme has not yet been established (Figure 6).

Sphingolipid metabolic diseases stemming from inborn error of metabolism such as Gauscher’s and Krabbe’s diseases, GM1 gangliosidosis, Tay-sach’s disease, etc. have already been extensively studied and reported [34–38]. In all these cases, it has been noted that there is an accumulation of a specific sphingolipid in a particular tissue(s)/organs due to the deficiency of a metabolic enzyme. However, no such disease relevant to Cer or Sph accumulation has yet been reported. Cer has been at the center of extensive study for its role in various cell death mechanisms in the nervous system disorders [11,29–31]. We have recently reported that Sph elevation has been preceded via transient accumulation of Cer in MS brains [15]. A major portion of Cer accumulation in MS tissues has been contributed by the cytokine stimulated serine palmityoltransferase activation, a pathway leading to Cer biosynthesis rather than sphingomyelin degradation (salvage pathway) (Figure 6). Although Sph has been shown to trigger cell death in oligodendrocytes (Figure 5), the precise mechanism for this cell death still remains unexplored.

In summary, we have shown that besides MGCs both Cer and Sph are necessary components for myelinogenesis and a defined ratio of Cer/dhCer as well as of Sph/dhSph is maintained during the rat brain development. In contrast, a great degree of variation of this ratio is observed in MS brain as both Cer and Sph at a higher than their normal biological concentrations lead to oligodendrocyte death and thereby demyelination.

## Abbreviations

Cer: Ceramide
dhSph: Dihydrosphingosine
GC-MS: Gas Chromatography and Mass Spectrometry
Gsl: Glycosphingolipid
HPLC: High Performance Liquid Chromatography
MGC: Monoglycosylceramide
MS: Multiple Sclerosis
Sph: Sphingosine
